# *IDH-*wild type glioblastomas featuring at least 30% giant cells are characterized by frequent *RB1* and *NF1* alterations and hypermutation

**DOI:** 10.1186/s40478-021-01304-5

**Published:** 2021-12-24

**Authors:** Valeria Barresi, Michele Simbolo, Andrea Mafficini, Maurizio Martini, Martina Calicchia, Maria Liliana Piredda, Chiara Ciaparrone, Giada Bonizzato, Serena Ammendola, Maria Caffo, Giampietro Pinna, Francesco Sala, Rita Teresa Lawlor, Claudio Ghimenton, Aldo Scarpa

**Affiliations:** 1 Department of Diagnostics and Public Health, Section of Anatomic Pathology, University of Verona, Verona, Italy; 2 Unit of Anatomic Pathology, Catholic University of Sacred Hearth, Rome, Italy; 3 ARC-NET Research Centre, University and Hospital Trust of Verona, Verona, Italy; 4 Department of Biomedical and Dental Sciences and Morphofunctional Imaging, Section of Neurosurgery, University of Messina, Messina, Italy; 5 Department of Neurosciences, Unit of Neurosurgery, Hospital Trust of Verona, Verona, Italy; 6 Department of Neurosciences, Biomedicines and Movement Sciences, Institute of Neurosurgery, University of Verona, Verona, Italy; 7 Department of Pathology and Diagnostics, University and Hospital Trust of Verona, Verona, Italy

**Keywords:** Giant cell, Glioblastoma, *RB1*, Mismatch repair, Tumor mutational burden

## Abstract

Giant cell glioblastoma (GC-GBM) is a rare variant of *IDH*-wt GBM histologically characterized by the presence of numerous multinucleated giant cells and molecularly considered a hybrid between *IDH*-wt and *IDH*-mutant GBM. The lack of an objective definition, specifying the percentage of giant cells required for this diagnosis, may account for the absence of a definite molecular profile of this variant. This study aimed to clarify the molecular landscape of GC-GBM, exploring the mutations and copy number variations of 458 cancer-related genes, tumor mutational burden (TMB), and microsatellite instability (MSI) in 39 GBMs dichotomized into having 30–49% (15 cases) or ≥ 50% (24 cases) GCs. The type and prevalence of the genetic alterations in this series was not associated with the GCs content (< 50% or ≥ 50%). Most cases (82% and 51.2%) had impairment in *TP53/MDM2* and *PTEN/PI3K* pathways, but a high proportion also featured *TERT* promoter mutations (61.5%) and *RB1* (25.6%) or *NF1* (25.6%) alterations. *EGFR* amplification was detected in 18% cases in association with a shorter overall survival (*P* = 0.004*)*. Sixteen (41%) cases had a TMB > 10 mut/Mb, including two (5%) that harbored MSI and one with a *POLE* mutation. The frequency of *RB1* and *NF1* alterations and TMB counts were significantly higher compared to 567 *IDH* wild type (*P* < 0.0001; *P* = 0.0003; *P* < 0.0001) and 26 *IDH*-mutant (*P* < 0.0001; *P* = 0.0227; *P* < 0.0001) GBMs in the TCGA PanCancer Atlas cohort. These findings demonstrate that the molecular landscape of GBMs with at least 30% giant cells is dominated by the impairment of *TP53/MDM2* and *PTEN/PI3K* pathways, and additionally characterized by frequent *RB1* alterations and hypermutation and by *EGFR* amplification in more aggressive cases. The high frequency of hypermutated cases suggests that GC-GBMs might be candidates for immune check-point inhibitors clinical trials.

**Supplementary Information:**

The online version contains supplementary material available at 10.1186/s40478-021-01304-5.

## Introduction

Glioblastoma (GBM) is classified into *Isocitrate Dehydrogenase* (*IDH)*-mutant and *IDH*-wild type (wt) [1]. The former mainly affects younger patients and has a better prognosis [2, 3].

Among *IDH*-wt GBMs, giant cell (GC)-GBM represents a rare histological variant, that accounts for less than 1% of all cases [4] and is histologically characterized by bizarre multinucleated giant cells [1]. It is reported to affect younger subjects and to have a relatively better prognosis compared to conventional *IDH*-wt GBM [5].

It is still unclear whether GC-GBM represents a distinct entity or only a morphological variant of *IDH*-wt GBM. Most of our current knowledge on its genetic features comes from few available molecular studies, mainly focusing on the analysis of selected genetic anomalies [6–11]. According to these, GC-GBM seems to be a hybrid between *IDH*-wt and *IDH*-mutant GBM. Similarly to the former, it has a high prevalence of *PTEN* mutations (18/58 cases, 31%), but alike the latter, it also shows a high incidence of *TP53* mutations (73/83 cases, 88%), low frequency of *EGFR* amplification (10/89 cases; 11%) and of *TERT* promoter mutations (21/65, 32%) [6–11]. Only one study performed a comprehensive molecular profiling of 10 GC-GBMs by whole exome sequencing [10]. In addition to confirming that GC-GBM has frequent impairment of *TP53/MDM2* (5 cases) and *PTEN/PI3K* (4 cases) pathways, it suggested that this morphological variant may be characterized by mutations in chromatin remodeling genes *SETD2* (3 cases) and *ATRX* (2 cases) and alterations in *RB1* (2 cases) [10]. Of note, one of the cases showed elevated tumor mutational burden (TMB) in association with *MSH6* somatic mutation [10], which may indicate that this is an additional, though exceptional, feature of this variant.

Based on its heterogeneous DNA-methylation profile, GC-GBM is not currently considered to represent a distinct molecular entity [12]. However, due to the lack of an objective definition, specifying the exact percentage of giant cells required for this diagnosis, the molecular portrait of GC-GBM is hardly definable. In a recent paper, the mutation frequencies of *TP53*, *ATRX*, *RB1*, and *NF1* were significantly higher in 17 GBMs featuring > 30% giant cells than in 357 *IDH*-wt GBMs in the TCGA PanCancer Atlas cohort [6].

In order to clarify the molecular landscape of GC-GBM, in this study we explored the mutations and copy number variation (CNV) of 458 cancer-related genes, microsatellite instability (MSI) and TMB, in 39 GBMs featuring at least 30% multinucleated giant cells and dichotomized into having 30–49% (15 cases) or ≥ 50% (24 cases) GCs.

## Materials and methods

### Cases

Thirty-nine formalin-fixed paraffin-embedded (FFPE) surgically resected and treatment *naïve* GBMs, featuring at least 30% multinucleated giant (i.e. having from few to more than 20 nuclei and a minimum diameter of 20 µm), bizarre (i.e. with atypical, hyperchromatic nuclei, and with evident nucleoli at times), with positive GFAP staining or not, were included in this study.

Taking as a reference the method proposed by Cantero et al*.* [6], the percentage of multinucleated giant cells was manually quantified by counting at least 1000 neoplastic cells in 10–20 random fields at 200 × magnification.

All cases were independently revised by three pathologists (VB, MM, CG), who assessed the percentage of giant cells. In case of disagreement, the cases were reviewed using a multi-headed microscope. The paraffin block with the highest number of GCs was selected for the subsequent molecular and immunohistochemical analyses.

Data on the overall survival (OS) were retrieved using clinical records.

### Ethics

This study was approved by the Local Ethics Committees of the Polyclinic A. Gemelli of Rome (protocol n. 1722, 2017/11/23) and of Verona (Protocol n. 35,628, 2020/06/29).

### Mutational and copy number variation status of cancer-related genes

Tumor mutational burden, mutations and copy number variations of 409 cancer-related genes were assessed using the targeted next generation sequencing (NGS) panel Oncomine Tumor Mutational Load (TML) (ThermoFisher), which covers 1.65 Mb of genomic space.

The results were confirmed using the SureSelectXT HS CD Glasgow Cancer Core assay (Agilent) in 29 GC-GBMs (cases 42GL-71GL).

DNA was obtained from 10 FFPE consecutive 4-μm sections using the QIAamp DNA FFPE Tissue Kit (Qiagen) and qualified as reported elsewhere [13].

Sequencing was performed on Ion Torrent platform using 20 ng of DNA for each multiplex PCR amplification and subsequent library construction. The quality of libraries was evaluated using the Agilent 2100 Bioanalyzer on-chip electrophoresis (Agilent Technologies). Libraries were clonally amplified by emulsion PCR with Ion OneTouch OT2 System (Thermofisher) and sequencing was run on Ion Proton (Thermofisher) loaded with Ion PI Chip v3.

Torrent Suite Software v.5.10 (Termofisher) was used for data analysis, including alignment to the hg19 human reference genome and variant calling. Filtered variants were annotated using a custom pipeline based on vcflib (https://github.com/ekg/vcflib), SnpSift [14], Variant Effect Predictor (VEP) [15] and NCBI RefSeq database. Additionally, alignments were visually verified with the Integrative Genomics Viewer (IGV) v2.9 [16] to confirm the presence of identified mutations. Germline mutations were assigned based on Sun et *al.* [17].

CNV was evaluated using OncoCNV v6.8 [18], comparing the BAM files obtained from tumor samples with those obtained from blood samples of four healthy males. The software includes a multi-factor normalization and annotation technique enabling the detection of large copy number changes from amplicon sequencing data and permits to visualize the output per chromosome.

### Confirmation of mutational and copy number variation status of 125 cancer-related genes and further exploration of 49 genes

Twenty-nine cases (42GL-71GL) were additionally analyzed using the SureSelectXT HS CD Glasgow Cancer Core assay (www.agilent.com), hereinafter referred as CORE [19] (details in Additional file 2). This spans 1.85 Mb of the genome and interrogates 174 genes (49 of which are not included in the TML panel) for somatic mutations, copy number alterations and structural rearrangements.

Sequencing libraries were prepared by targeted capture using the SureSelect kit (Agilent Technologies) according to the manufacturer instructions as previously described [20]. Genomic DNA was enzymatically fragmented with the SureSelect Enzymatic Fragmentation Kit (Agilent Technologies). Quality and quantity of pre-capture libraries was assessed using the Qubit BR dsDNA assay (ThermoFisher). Hybridization-capture and purification of the libraries was performed using 100 ng from each pre-capture library to prepare 16-library pools (1.6 µg of total pooled DNA). Captured library pools were enriched by PCR, purified, and quantified using the Qubit dsDNA HS assay. Quality of the library pools was verified with the Agilent 4200 Tape Station and High Sensitivity D1000 ScreenTape (Agilent Technologies). Sequencing was performed on a NextSeq 500 (Illumina) loaded with 2 captured library pools, using a high-output flow cell and 2 × 75 bp paired-end sequencing.

CORE panel analysis was performed as previously described [20]. Briefly, demultiplexing was performed on the BaseSpace Sequence Hub (https://basespace.illumina.com). Paired-end reads were aligned to the human reference genome (version hg38/GRCh38) using BWA and saved in the BAM file format [21]. BAM files were sorted, subjected to PCR duplicate removal, and indexed using biobam-bam2 v2.0.146 [22]. Coverage statistics were produced using samtools [23]. Single nucleotide variants were called using Shearwater [24]. Small (< 200 bp) insertions and deletions were called using Pindel [25]. Small nucleotide variants were further annotated using a custom pipeline based on vcflib (https://github.com/ekg/vcflib; last access 11/30/2020), SnpSift [14], the Variant Effect Predictor (VEP) software [15], and the NCBI RefSeq transcripts database (www.ncbi.nlm.nih.gov/refseq/). Annotated variants were filtered keeping only missense, nonsense, frameshift, or splice site variants. All candidate mutations were manually reviewed using Integrative Genomics Viewer (IGV), version 2.9 [16] to exclude sequencing artefacts. Gene copy number alterations were detected using the geneCN software (https://github.com/wwcrc/geneCN). Whole-chromosome or chromosome-arm alterations were assessed by measuring the ratio of normalized, GC-adjusted coverage of tumor samples’ alignments to the mean, normalized, GC-adjusted coverage of 20 non-neoplastic samples for all targeted regions of a chromosome arm. Targeted regions included both targeted genes and a set of “backbone” regions probing each chromosome at 1 megabase intervals. Each large alteration was further confirmed by checking the copy number status of targeted genes included in the large alteration itself as reported by the geneCN software.

### Classification of genetic variants

Following the five-tier classification system recommended by the joint consensus of the American College of Medical Genetics and Genomics and the Association for Molecular Pathology (ACMG/AMP)[26], variants were classified: Benign (class 1); Likely Benign (class 2); Variant of Un-certain Significance (VUS – class 3); Likely Pathogenic (class 4); Pathogenic (class 5). Variants’ classification was retrieved from the ClinVar database when available (https://www.ncbi.nlm.nih.gov/clinvar/) and accepted when the record complied with the following requisites: reviewed by expert panel according to the ACMG/AMP guidelines and/or reported by multiple submitters with evaluation criteria according to the ACMG/AMP guidelines and no conflicts. When a consistent classification was unavailable or when the variant was not present in the ClinVar database, variants were evaluated in-house, according to the ACMG/AMP guidelines using also the following databases and software to gather and integrate all relevant information: My Cancer Genome (https://www.mycancergenome.org), Intogen [27] (https://www.intogen.org/) and QIAGEN Clinical Insight (QCI) software (https://variants. qiagenbioinformatics.eu/qci/).

### *TERT* promoter mutational analysis

*TERT* was amplified by PCR and both strands were sequenced using the ABI PRISM 3500 Genetic Analyzer (Applied Biosystems) as previously described [28]. The primers used were: TERT-F GTCCTGCCCCTTCACCTT and TERT-R GCACCTCGCGGTAGTGG.

### Tumor mutational burden

TMB and mutational spectrum were evaluated using the Oncomine TML 5.10 plugin available on IonReporter software (Thermofisher). Default Modified parameters were used to exclude sequencing artefacts. In detail, a threshold of at least 60 reads and 10% allelic frequency was used for variant calling. TMB was expressed as the number of mutations per Mb (muts/Mb), where mutations included nonsynonymous missense and nonsense single nucleotide variants (SNVs) detected per Mb of exonic sequences.

### Immunohistochemistry of DNA mismatch repair proteins

Immunostaining was performed using the Bond Polymer Refine Detection kit (Leica Biosystems) in a BOND-MAX system (Leica Biosystems) on 4 μm-thick FFPE sections using the following primary antibodies purchased from DakoCytomation: mouse monoclonal clones ES05 against MLH1 (dilution 1:30) and FE11 against MSH2 (dilution 1:30); rabbit monoclonal clones EP49 against MSH6 (dilution 1:100) and EP51 against PMS2 (dilution 1:100). Normal cells within the samples acted as positive internal controls.

### Microsatellite instability analysis

MSI was tested by a fluorescent multiplex PCR exploiting the 5 mononucleotide microsatellites BAT25, BAT26, NR21, NR22, NR24. Amplicons were separated by capillary electrophoresis using the ABI Genetic Analyzer 3130XL (Applied Biosystems). Variations ≥ 3 bp for BAT25, NR21, NR22, NR24 and ≥ 4 bp for BAT26 were considered as instability.

### Comparison with GBMs *IDH*-wt and *IDH*-mutant in The Cancer Genome Atlas database

In order to compare the clinical and genetic findings in this cohort of GBMs with giant cells with those in *IDH*-wt and *IDH*-mutant GBMs, we accessed The Cancer Genome Atlas (TCGA) databases for GBMs (cbioportal.org) and retrieved data from the series of “Glioblastoma Multiforme (TCGA PanCancer Atlas)”.

### Statistical analysis

We used Chi-squared and Mann–Whitney tests to analyze the correlation between the percentage of giant cells or TMB and the various genetic alterations, and to assess the statistical difference in the patients age, frequency of genetic alterations or in TMB between the present 39 GBMs with giant cells and *IDH*-wt or *IDH*-mutant GBMs in TCGA PanCancer Atlas.

Overall survival (OS) of the patients was assessed by the Kaplan–Meier method, using the date of surgery as the entry data and the length of survival until the patient’s death as the endpoint. Patients who died of GBM-independent diseases were censored. Mantel–Cox log-rank test was applied to assess the strength of association between OS and each variable. Successively, a multivariate analysis (Cox regression model) was utilized to determine the independent effect of the variables on OS.

Mantel-Cox log-rank test was also carried out to analyze the difference in the OS of patients win this cohort with and those with *IDH*-wt or *IDH*-mutant GBM in TCGA PanCancer Atlas.

A *P*-value < 0.05 was considered as significant. All analyses were performed using MedCalc for Windows version 15.6 (MedCalc Software, Ostend, Belgium) and R v.3.2.1.

## Results

### Cases

The clinical-pathological features of the 39 GBMs are summarized in Additional file 3: Table 1.

Male to female ratio was 2:1 (26 male and 13 female patients) and median age was 63 years (mean age: 57.6 years, range 15–84). Fifteen patients were < 55 years, while 24 were ≥ 55 years. All tumors were localized in the brain lobes, except for 3 cases that were in the third ventricle. All patients had surgery, followed by chemotherapy with temozolomide and radiotherapy.

**Fig. 1:**
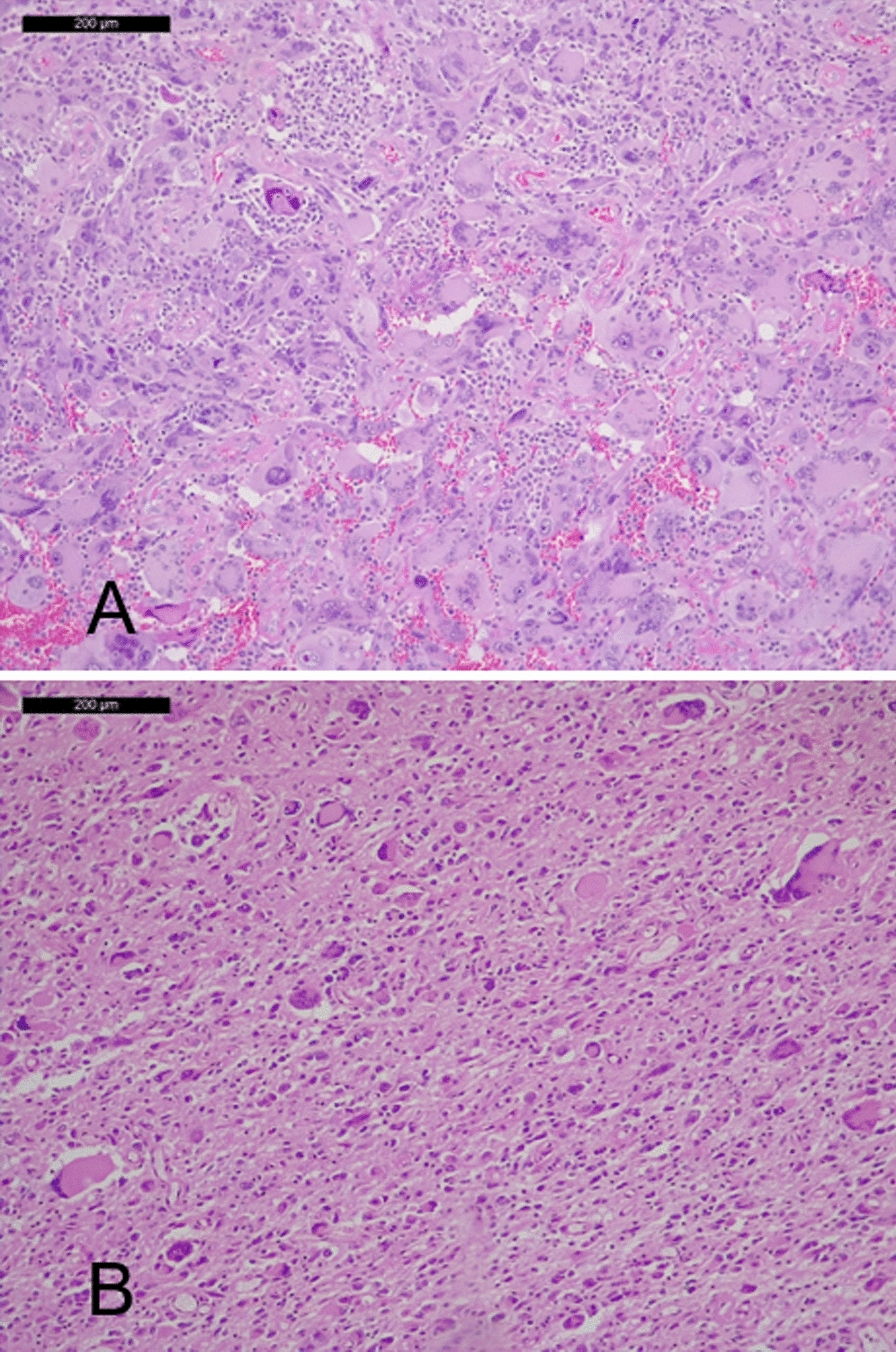
Histological aspect of giant cell enriched glioblastomas. In the upper image is a glioblastoma classified as having ≥ 50% giant cells, while in the lower is a glioblastoma classified as having 30–49% giant cells

All tumors featured frequent atypical mitoses. Thirty-five had microvascular proliferation and 33 had necrosis. The percentage of GCs ranged between 30 and 90%.The cases were dichotomized into having 30–50% GCs (13 cases) and ≥ 50% GCs (22 cases) (Fig. 1).

### Mutational status of 458 genes

**Fig. 2:**
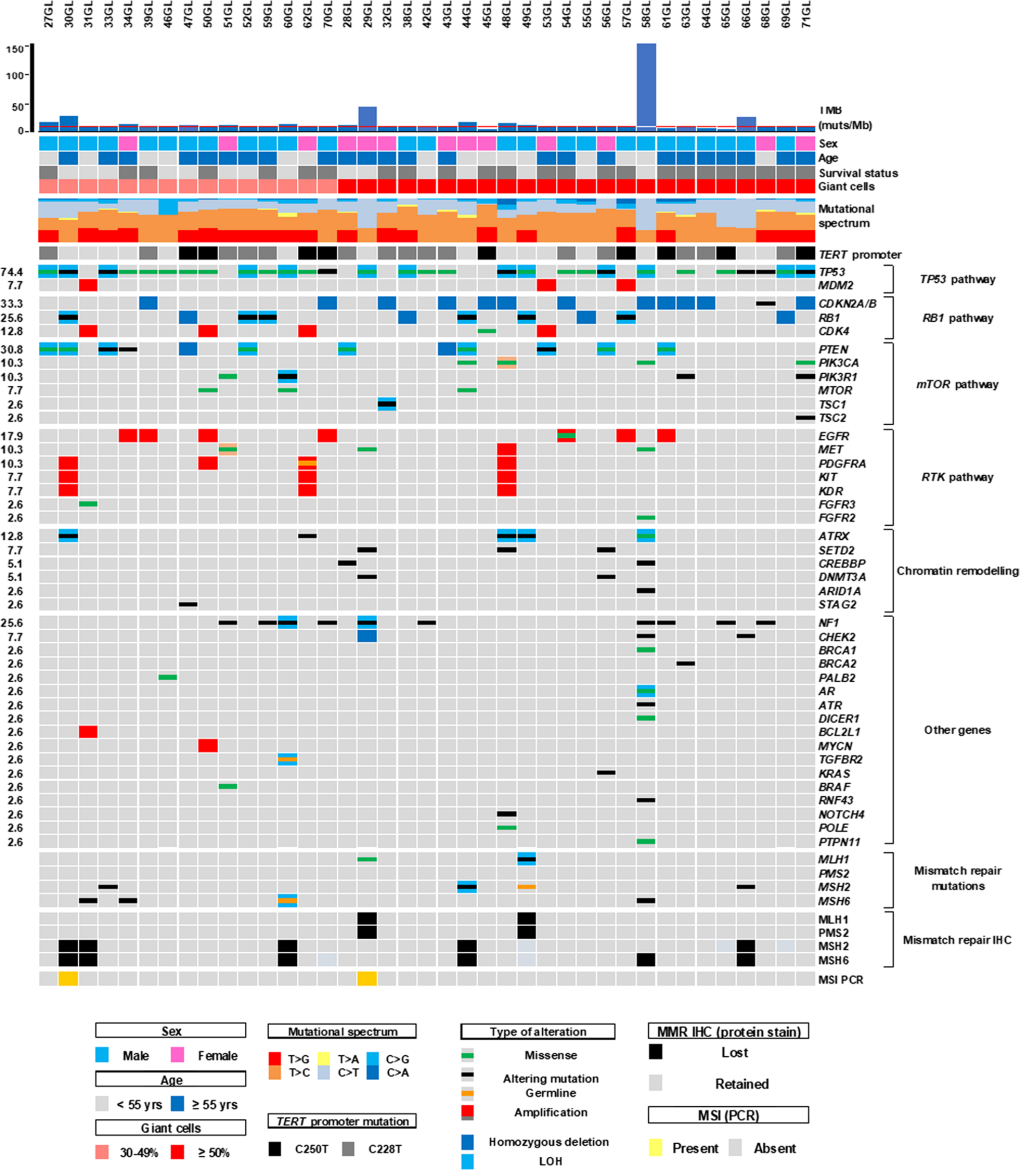
**Clinical-pathological features, gene alterations and MMR status of 39 giant cells enriched GBMs.** The matrix shows for each case the tumor mutational burden, mutational signature, gene alterations, immunohistochemical analysis of genes involved in DNA mismatch repair (MMR IHC) and the presence of microsatellite instability as assessed by MSI-PCR. Samples are sorted by the percentage of giant cells (30–49%; ≥ 50%) and then by ID number. Genes are grouped by pathway and then by frequencies of alterations and alphabetical order

The alterations in 409-cancer related genes, detected in the 39 GBMs using TML and CORE panels, and those in additional 49 genes using the CORE panel in a subset of 29 cases (42GL-71GL) are summarized in Fig. 2, detailed in Additional file 3: Table 2 and described below according to the altered pathway. Genes’ mutations and CNV were not significantly different according to the percentage of GCs. Regarding the 125 genes in common between the two panels, the CORE confirmed the presence of the alterations identified using the TML panel in the subgroup of 29 cases (cases 42GL-71GL).

### *IDH1/2* mutations

None of the cases had *IDH1/2* mutations.

### *TP53/MDM2* pathway

Thirty-two GBMs (82%) had alterations in p53 pathway. In detail, 29 (74.4%) cases had *TP53* mutations, that co-occurred with *CDKN2A* homozygous deletion in 8. Among *TP53* wild type tumors, three had *MDM2* amplification and 4 had *CDKN2A* homozygous deletion.

### *RB1/CDKN2A/CDK4* pathway

Twenty-seven (69%) GBMs had alterations. Ten (24.6%) had *RB1* inactivation due to homozygous deletion (4 cases), or heterozygous deletion combined with mutation of the other allele (6 cases). Among the cases with intact *RB1*, four had *CDK4* amplification, 12 featured the homozygous deletion of *CDKN2A/B* and one had a truncating mutation of *CDKN2A*.

### *PI3K//PTEN/AKT/mTOR* pathway

Twenty (51.2%) GBMs had alterations in this pathway. Twelve had *PTEN* alterations consisting in mutations (1 case), homozygous deletion (2 cases) or heterozygous deletion combined with mutation of the other allele (9 cases). In one case, *PTEN* alteration co-occurred with *PIK3CA* and *mTOR* mutations. Of the *PTEN* wild type cases, two had *PIK3CA* mutations, one had co-occurring *PIK3CA*, *PIK3R1* and TSC2 mutations, three had *PIK3R1* mutation, associated with *mTOR* mutation in one case, one had *mTOR* mutation, one had *TSC1* heterozygous deletion coupled with the mutation of the second allele.

### Receptor Tyrosine Kinase pathway

Thirteen (33.3%) GBMs had activation of Receptor Tyrosine Kinase signaling pathways. Seven cases (17.9%) had *EGFR* amplification, co-occurring with *PDGFRA* amplification (50GL) in one case. Of the *EGFR* unamplified cases, three showed the concurrent amplification of *PGFRA*, *KIT* and *KDR*, three had *MET* mutations, with co-occurring *FGFR2* mutation in one case, and one had *FGFR3* mutation.

### Chromatin remodeling pathway

Eight (20.5%) GBMs had alterations in chromatin remodeling genes, including *ATRX* (5/39; 12.8%), *ARID1A* (1/39; 2.6%), *SETD2* (3/39; 7.6%), *CREBBP* (2/39; 5.1%), *DNMT3A* (2/39; 5.1%).

### MMR genes

Nine (23%) GBMs had sequence alterations in *MMR* genes. Three had somatic mutations of *MSH2*, three featured somatic mutations of *MSH6* and one had a somatic mutation of *MLH1*. One additional case had concurrent somatic mutation of *MLH1* and germinal mutation of *MSH2* and another had a germinal mutation of *MSH6*.

### Other genes

GBMs featured mutations in other genes, among which *NF1* was the most frequently mutated (10/39; 25.6%). Of note, one case (48GL) had *POLE* mutation.

### *TERT *promoter

Twenty-four (61.5%) GBMs had *TERT* promoter mutations. Fifteen (38.5%) had C228T mutation and 9 (23%) had C250T mutation. In one case (62GL) *TERT* promoter mutation C250T co-occurred with *ATRX* mutation.

### Numerical chromosomal alterations

Based on the chromosomal position of each gene, the status of chromosome arms was inferred. The most frequent chromosomal alterations were gains of chromosome 7 (15/39; 38.5%) and loss of chromosome 10 (23/39, 74.3%) (Additional file 1: Fig. 1).

### Tumor mutational burden

The number of mutations/Mb ranged between 5.4 and 153.8 (median: 9.3; inter-quartile range:8.2–12) (Fig. 1, Table 1). Using the cut-off of 10 mutations/Mb by Campbell et al. to define hypermutation [29], sixteen (41%) GC-GBMs were hypermutated.

**Table 1:** Univariate and multivariate analyses for OS in 39 patients with giant cells enriched GBM

Parameter	n	Univariate analysis		Multivariate analysis	
		H.R. (95% C.I.)	*P*	H.R. (95% C.I.)	*P*
*Age*					
< 55 years	15	1		1	
≥ 55 years	24	2.7 (1.1–6.2)	0.019	0.2 (0.1–0.7)	0.0117
*Sex*					
M	26	1			
F	13	2.6 (0.9–7.2)	0.062		
*% Giant cells*					
30–49%	15	1			
≥ 50%	24	1.7 (0.7–4)	0.205		
*TP53* mutations					
No	10	1			
Yes	29	0.3 (0.1–1.2)	0.116		
*RB1* mutations					
No	29	1			
Yes	10	0,5 (0.2–1.4)	0.215		
*PTEN* mutations					
No	27	1			
Yes	12	1.7 (0.5–5.1)	0.331		
* CDKN2A/B* homozygous deletion					
No	27	1			
Yes	12	2.2 (0.8–5.5)	0.086		
*EGFR amplification*					
No	32	1		1	
Yes	7	6.5 (1.7–24)	0.004	3.6 (1.4–9.3)	0.007
*NF1* mutations					
No	29				
Yes	10	0.5 (0.2–1.4)	0.228		
*TERT* promoter mutations					
No	15				
Yes	24	2.2 (0.9–5.3)	0.055		
Chromosome 7 gains					
No	24				
Yes	15	1.9 (0.7–4.6)	0.152		
Chromosome 10 LOH					
No	16				
Yes	23	1.1 (0.4–2.7)	0.753		
Hypermutation					
No	23	1		1	
Yes	16	0.3 (0.1–0.8)	0.0263	0.3 (0.1–0.8)	0.018

H.R:: hazard ratio. C.I.: confidence interval

Cases with *TERT* promoter mutation had significantly lower TMB (median TMB: 8.8 mutations/Mb) than cases with wild-type *TERT* promoter (median TMB: 13.1 mutations/Mb) (*P* = 0.0061). One hypermutated GC-GBM (48GL) had a *POLE* mutation.

### Microsatellite Instability

Two (5.1%) cases had MSI (29GL; 30G) as assessed by the PCR analysis of mononucleotide microsatellites (Additional file 3: Table 3; Fig. 2).

### MMR protein immunohistochemistry

**Fig. 3:**
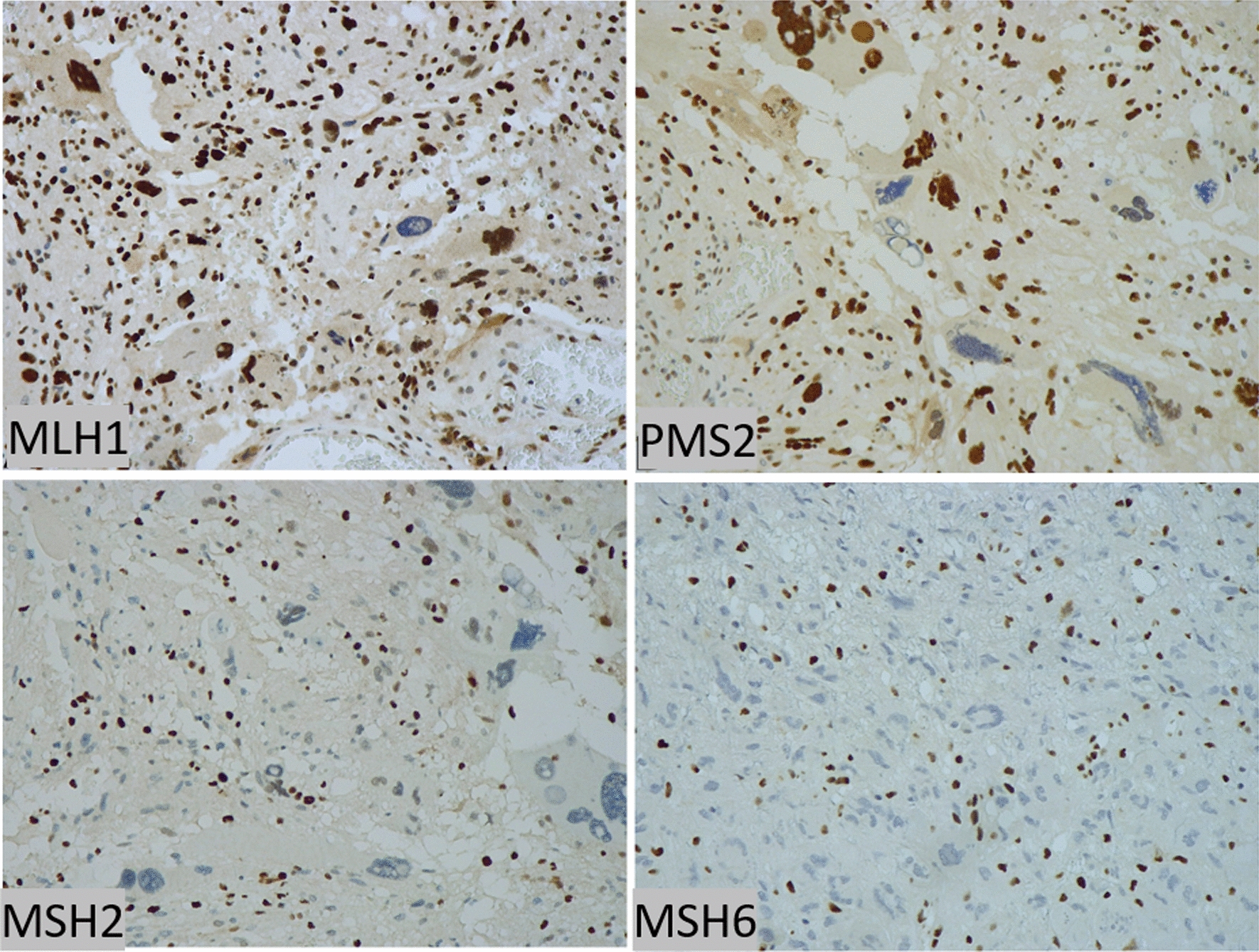
**Immunostaining of MMR proteins in a GBM enriched in GCs.** This case showed the loss of MSH2 and MSH6 in all tumor cells (60GL), albeit having stable microsatellites

Immunostaining of MMR proteins was classified as retained or lost (when absent in all tumor cells). Loss of MMR protein immunostaining was found in 8 cases, including 7 with the concordant loss of the matched pair partners (MSH2/MSH6 or MLH1/PMS2) and one with loss of MSH6 only (case 58GL) (Fig. 1; Additional file 3: Table 3). Namely, the concordant loss of MSH2/MSH6 was found in 5 cases (30GL, 31GL, 44GL, 60GL, 66GL) (Fig. 3), while that of MLH1/PMS2 was found in 2 (29GL, 49GL).

### Correlation of MMR immunohistochemistry, MSI status and MMR gene mutations and TMB

Of the 8 cases with MMR protein losses, only 2 featured MSI, while 5 with concordant losses and the case with loss of MSH6 only had stable microsatellites (Fig. 1; Additional file 3: Table 3).

Of the 2 cases with MSI, one had *MMR* gene mutations (29GL), while the other case (30GL) had no *MMR* gene mutations. Of the 37 cases with stable microsatellites, 8 showed *MMR* gene mutations. These included 2 with retained MMR proteins, 1 with concordant loss of MLH1/PMS2, four with concordant loss of MSH2/MSH6 and 1 with loss of MSH6 (Additional file 3: Table 3).

Of the 16 hypermutated cases, 2 had MSI and matched loss of MSH2/MSH6 proteins or MLH1/PMS2, 5 had stable microsatellites and the matched loss of MSH2/MSH6 (4 cases) or of MLH1/PMS2 (1 case), 1 had stable microsatellites and the isolated loss of MSH6 protein and 9 had stable microsatellites and no MMR loss.

### Survival analysis

Information on the OS was available for all patients. At the last follow-up time, 15 patients were alive and 24 had died of GBM. OS ranged between 4 and 27 months for died patients, while follow-up time ranged between 2 and 72 months for alive patients (Additional file 3: Table 1).

At univariate analyses, we tested the effect on patients’ survival of the following variables: age (< 55 years vs ≥ 55 years); sex; percentage of GCs (30–49% vs ≥ 50%); mutation in *TP53, NF1* or *TERT* promoter; alteration of *RB1* or *PTEN*; homozygous deletion of *CDKN2A/B;* amplification of *EGFR;* hypermutation; gains of chromosome 7; loss of chromosome 10.

**Fig. 4:**
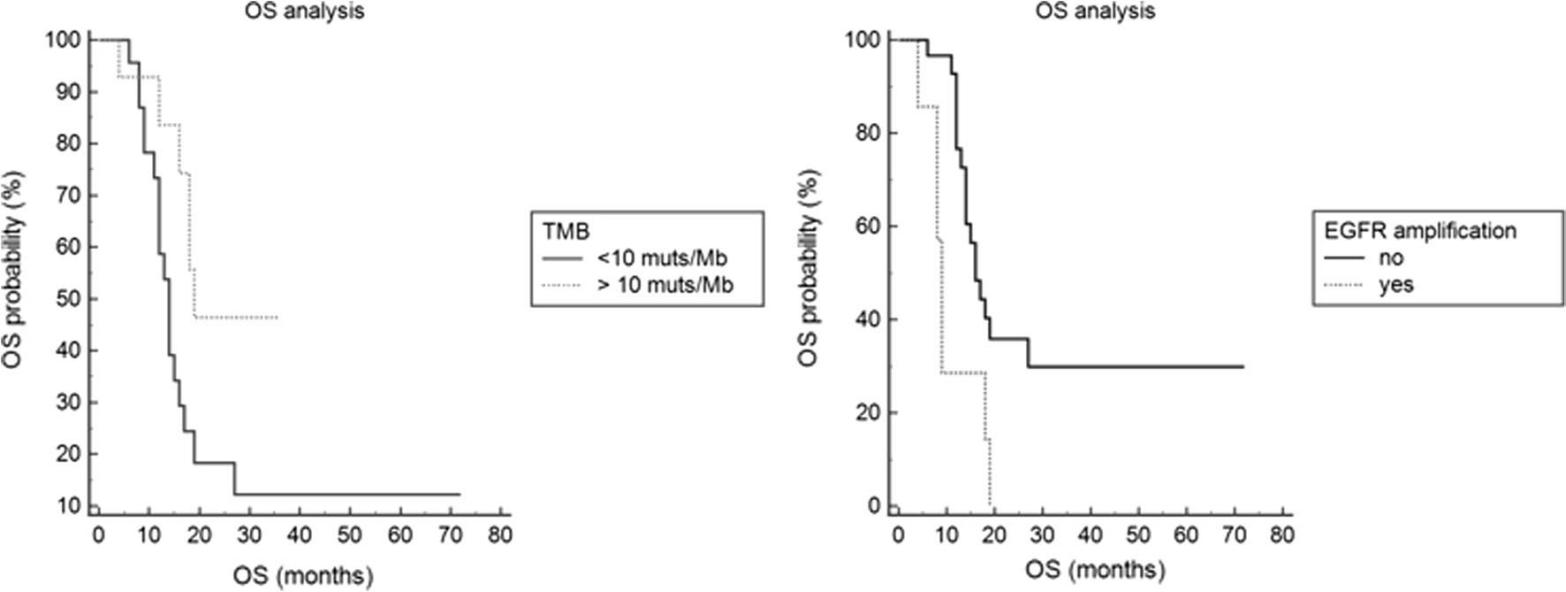
Impact of *EGFR* amplification and hypermutation on clinical outcome. The overall survival of patients with glioblastomas enriched in giant cells and harboring *EGFR* amplification (*P* = 0.004), or TMB < 10 mutations/Mb (*P* = 0.0263) was significantly shorter than that of patients with giant cells enriched glioblastoma lacking *EGFR* amplification or having TMB ≥ 10 mutations/Mb

Age ≥ 55 years (*P* = 0.019; Hazard Ratio: 2.7; 95%CI: 1.1–6.2) and *EGFR* amplification (*P* = 0.004; Hazard Ratio: 6.5; 95%CI: 1.7–24) were significantly associated with shorter OS (Table 1; Fig. 4). The presence of hypermutation (*P* = 0.0263; Hazard Ratio: 0.3; 95%CI: 0.1–0.8) was significantly associated with longer OS (Table 1; Fig. 3).

Multivariate analysis, including age of the patients, *EGFR* amplification and hypermutation as covariates, showed that all three were independent prognostic variables (Table 1).

### Comparison of the present GBM series with the TCGA PanCancer Atlas GBM series

To clarify whether GBMs featuring > 30% GCs are a distinct group, we compared their clinical features, TMB and genes mutations/CNV with those of 567 *IDH*-wt and 26 *IDH*-mutant GBMs in TCGA PanCancer Atlas series.

The age of the patients in the present series was significantly higher than that of the patients with *IDH*-mutant GBMs (*P* = 0.0001), but not different from that of the patients with *IDH*-wt GBM (*P* = 0.440) (Table 2).

**Table 2:** Comparison between the genetic alterations and TMB in the present 39 GBMs and in TGCA (PanCancer Atlas cohort) *IDH*-wt and *IDH*-mutant GBMs

	Present GBMs (n = 39)		TGCA *IDH-*wt GBMs*		*P*	TGCA *IDH*-mutant GBMs**		***P***
*Clinical features*								
Mean age; age range	57.6 years; 15–84 years		60 years; 10–89 years		0.440	38 years; 21–60 years		**0.0001**
Male:Female	2:1		1.4:1			1.5:1		
TMB ***								
median; range; inter-quartile range	9.3 muts/Mb; 5.4–153.8; 8.2–12		1.7 muts/Mb; 0–230; 1.4–2.2		< 0.0001	1.4 muts/Mb; 0.6–405; 1.1–2		** < 0.0001**
MSI****	2	5.1%	3	1.6%	0.210	0	0	0.512
*Genetic alterations*	n	%	n	%		n	%	
*TP53*	29	74.4%	100	27.0%	** < 0.0001**	25	96.2%	**0.022**
*PTEN*	12	30.8%	187	33.9%	0.686	1	4.2%	** < 0.0001**
*CDKN2A/B* hom del	12	30.8%	318	57.7%	**0.0011**	4	16.7%	0.215
*RB1*	10	25.6%	52	9.4%	** < 0.0001**	1	4.2%	** < 0.0001**
*NF1*	10	25.6%	45	12.1%	**0.0003**	1	3.8%	**0.0227**
*EGFR* ampl	7	17.9%	255	46.3%	**0.0006**	0	0.0%	**0.0001**
*CDK4* ampl	5	12.8%	76	13.8%	0.864	6	25.0%	0.219
*ATRX*	5	12.8%	17	4.6%	**0.0301**	20	76.9%	** < 0.0001**
*MSH6*	4	10.3%	6	1.6%	**0.0009**	1	3.8%	0.345
*PIK3CA*	4	10.3%	35	9.4%	0.867	5	19.2%	0.308
*PIK3R1*	4	10.3%	34	9.2%	0.823	3	11.5%	0.871
*PDGFRA*ampl	4	10.3%	73	13.2%	0.592	2	8.3%	0.802
*MSH2*	4	10.3%	0	0.0%	** < 0.0001**	1	3.8%	0.345
*MDM2* ampl	3	7.7%	47	8.5%	0.856	0	0.0%	0.167
*MTOR*	3	7.7%	5	1.3%	**0.0007**	1	3.8%	0.530
*KIT* ampl	3	7.7%	54	9.8%	0.667	2	8.3%	0.927
*KDR*ampl	3	7.7%	35	6.4%	0.742	2	8.3%	0.0927
*SETD2*	3	7.7%	9	2.4%	0.0638	2	7.7%	1
*MLH1*	2	5.1%	1	0.3%	**0.0007**	0	0.0%	0.244
*CREBBP*	2	5.1%	6	1.6%	0.132	1	3.8%	0.810
*DNMT3A*	2	5.1%	2	0.5%	**0.0056**	1	3.8%	0.810
*MET* ampl	1	2.6%	14	2.5%	0.992	1	4.2%	0.726
*ARID1A*	1	2.6%	4	1.1%	0.421	1	3.8%	0.771
*TSC1*	1	2.6%	3	0.8%	0.289	1	3.8%	0.771
*TSC2*	1	2.6%	1	0.3%	0.0507	0	0.0%	0.414
*EGFR* mutation	1	2.6%	91	24.5%	**0.0018**	3	11.5%	0.143
*FGFR3*	1	2.6%	2	0.5%	0.158	1	3.8%	0.771
*FGFR2*	1	2.6%	4	1.1%	0.421	0	0.0%	0.414
*ARID2*	1	2.6%	1	0.3%	0.0507	1	3.8%	0.771

^*^371 samples were profiled for mutations and 551 for copy number variations (CNV)

^**^26 samples were profiled for mutations and 24 for CNV

^***^Mutation count was available for 368 *IDH*-wt and 26 *IDH*-mutant GBMs in TGCA PanCancer Atlas cohort

^****^MSI sensor score was available for 184 IDH-wt and 26 IDH-mutant GBMs in TGCA PanCancer Atlas cohort

genetic alterations are arranged by their frequency in the cohort of giant cells enriched GBMs. The statistical difference in the frequency of each genetic alteration between giant cells enriched GBMs and *IDH*-wt or *IDH*-mutant GBMs was assessed using Chi-squared test. The statistical difference in TMB was assessed using Mann–Whitney test

GBMs with > 30% giant cells had significantly higher TMB than both *IDH*-wt and *IDH*-mutant GBMs in TCGA (*P* < 0.0001). TMB was calculated in TCGA cases profiled using whole exome sequencing considering that an exome is 1% of the genome (i.e., 30 × 10^6^ bp).

In 567 *IDH*-wt GBMs, TMB ranged between 0 and 230 mutations/Mb with a median of 1.7 mutations/Mb (interquartile range 1.4–2.2) (Table 2). Ten (2.7%) cases had a TMB ≥ 10 mutations/Mb, including one (TCGA-19–5956 with TMB of 230 mutations/Mb) with *POLE* and *MLH1* mutations and two (TCGA-16–0848 with TMB of 11 mutations/Mb; TCGA-16–0829 with TMB of 20.3 mutations/Mb) with *MSH6* mutations. The review of the pathological reports of these cases showed that the *POLE*-mutated was a GC-GBM. Only one hypermutated case (TCGA-19–1787 with TMB of 17.2 mutations/Mb) had MSI, as defined by MSI sensor score ≥ 3.5[30]. Two other cases, including one (TCGA-06–0187) with a TMB of 1.3 mutations/Mb and another (TCGA-12–0772) with unavailable mutation count, had MSI sensor score ≥ 3.5.

In 26 *IDH*-mutant GBMs, TMB ranged between 0.6 and 405 mutations/Mb, with a median of 1.4 mutations/Mb (interquartile range 1.4–2.2). Two cases (7.6%) had a TMB ≥ 10 mutations/Mb, including one (TCGA-06–5416 with TMB of 405.9 mutations/Mb) with *POLE*, *MSH2* and *MSH6* mutations. None of the cases had MSI (MSI sensor score < 3.5).

In the TCGA series, 551 *IDH*-wt and 24 *IDH*-mutant GBMs were profiled for CNV; 371 *IDH*-wt and all 26 *IDH*-mutant GBMs were profiled for gene mutations.

GBMs with > 30% GCs had significantly higher frequency of *RB1* (*P* =  < 0.0001) and *NF1* alterations (*P* = 0.0003; *P* = 0.0227) than both *IDH*-wt and *IDH*-mutant GBMs in TCGA PanCancer Atlas.

In addition, they featured frequencies of *TP53* and *ATRX* mutations (74.4%; 12.8%) significantly higher than *IDH*-wt (27%; 4.6%; *P* < 0.0001; *P* = 0.0301) and lower than *IDH*-mutant GBMs (96.2%; 76.2%; *P* = 0.0139; *P* < 0.0001), and frequency of *EGFR* amplification (17.9%) significantly lower than *IDH*-wt (46.3%; *P* = 0.0006) and higher than *IDH*-mutant GBM (0%; *P* = 0.0001) (Table 2). The frequency of *PTEN* alterations was similar to that in *IDH*-wt GBMs (30.8% vs 33.9%; *P* = 0.686) and significantly lower than that in *IDH*-wt GBMs (4%; *P* = 0.0011).

Frequencies of alterations in *mTOR*, *MMR* and chromatin remodeling *DNMT3A* genes were similar to those in *IDH*-mutant GBMs, but significantly more frequent than those in *IDH*-wt GBMs (Table 2).

**Fig. 5:**
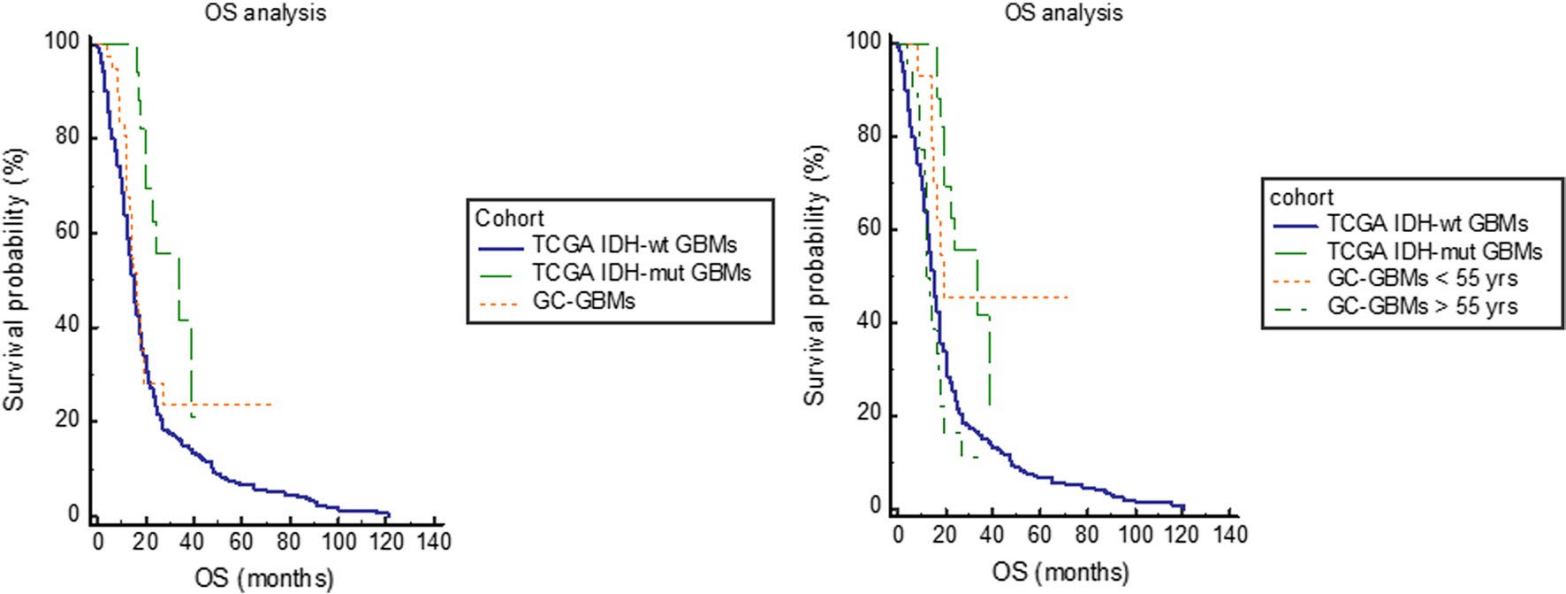
Comparison in the OS of patients with GBMs enriched in giant cells and TCGA GBMs *IDH*-wt or *IDH*-mutant. Patients with giant cells enriched GBM had an OS significantly shorter than patients with *IDH*-mutant GBM (*P* = 0.0127), but not significantly different from that of patients with *IDH*-wt GBM (*P* = 0.187). Patients with giant cell enriched GBM and younger than 55 years had an OS significantly longer than patients with *IDH*-wt GBM and similar to patients with *IDH*-mutant GBM, while those of 55 years or older had an OS length similar to *IDH*-wt GBM and significantly shorter than *IDH*-mutant GBM (*P* = 0.0013)

The OS was known for 568 patients with *IDH*-wt (median: 12 months; range: 0–121 months; 440 died of GBM) and 24 with *IDH*-mutant (median: 20 months; range: 3–41 months; 9 died of GBM) GBMs in TCGA PanCancer Atlas series. Patients with GBMs in this series had an OS length significantly shorter than patients with *IDH*-mutant GBM (Hazard ratio: 0.4; 95% C.I.: 02–0.8; *P* = 0.0127), but not significantly different from patients with *IDH*-wt GBM (Hazard ratio: 1.3; 95% C.I.: 0.9–1.8; *P* = 0.187) (Fig. 5).

When the GC-GBMs in this cohort were dichotomized on the basis of the age at diagnosis (< 55 years and ≥ 55 years), the patients younger than 55 years had on OS longer than patients with *IDH*-wt GBMs (Hazard ratio: 0.4; 95% C.I.: 0.2–0.7) and similar to patients with *IDH*-mutant GBMs (Hazard ratio: 1.2; 95% C.I.: 0.6–2.2) in TCGA PanCancer Atlas series (*P* = 0.0013) (Fig. 5). On the other hand, the patients of 55 years or older had an OS significantly shorter than patients with *IDH*-mutant GBMs (Hazard ratio: 3.1; 95% C.I.: 1.6–6) and similar to patients with *IDH*-wt GBMs (Hazard ratio: 1.1; 95% C.I.: 0.6–1.8) in TCGA PanCancer Atlas series (*P* = 0.0013) (Fig. 5).

## Discussion

The 2016 WHO classification defines GC-GBM as a variant of *IDH*-wt GBM characterized histologically by numerous multinucleated giant cells and molecularly by a high frequency of *TP53* mutations and rare *EGFR* amplification [1].

In this study on 39 GBMs featuring a percentage of giant cells ranging between 30 and 90%, the alterations found in 458 cancer-related genes analyzed with NGS were not associated with the giant cell content (30% -50% or > 50%). As expected, no cases had *IDH1/2* mutations and a high percentage (82%) featured alterations of *TP53/MDM2* pathway*.* However, a consistent proportion (69.2%) of GC-GBMs also harbored alterations in *RB1/CDKN2A/CDK4* pathway, with 25.6% cases having impairment of *RB1*, 33.3% displaying *CDKN2A* homozygous deletion and 10% showing *CDK4* amplification. *EGFR* amplification was found in 18% cases and was significantly correlated to a worse prognosis. Other frequent alterations were detected in *NF1* (25.6%), chromatin remodeling genes (25.6%) (including 12.8% mutations in *ATRX* and 7.6% in *SETD2*), and MMR genes (23%). The comparison with GBMs in the TCGA PanCancer Atlas cohort revealed that the rates of *TP53* and *ATRX* mutations, *PTEN* alterations, *EGFR* amplification and *CDKN2A/B* homozygous deletion in the present series were intermediate between those found in *IDH*-wt and *IDH*-mutant GBMs. In contrast, the frequency of *RB1* or *NF1* (25.6%) alterations was significantly higher than in both TCGA groups (14% vs 3.8%, for *RB1*; 12.1 vs 3.8% for *NF1*), suggesting that this is a distinctive feature of GBMs enriched in GCs. In accordance, 2/10 (20%) GC-GBMs analyzed in a previous study by whole exome sequencing had *RB1* mutations [10], and 8 (47%) and 6 (35%) of 17 GBMs with > 30% giant cells had *RB1* and *NF1* mutations in another [6].

One of the present GBMs had a pathogenic *POLE* mutation, similarly to other reported cases of GBMs enriched in giant cells [6, 28, 31, 32], which suggests that also *POLE* mutations may be part of the molecular portrait of GC-GBM.

Therefore, our findings confirm and expand the concept that GC enriched GBM is a peculiar entity, distinct from either *IDH*-wt or *IDH*-mutant GBM. In most cases (32 cases; 82%), it is driven by the alteration of P53 function due to either *TP53* gene mutations (29 cases, 74.4%) or amplification of its principal cellular antagonist, the *MDM2* gene (3 cases, 7.7%). However, it is also enriched in alterations of *RB1/CDKN2A/CDK4* pathway and mutations in *NF1*, *POLE*, and chromatin remodeling genes.

A major issue in the diagnosis of GC-GBM is represented by the lack of a cut-off of giant cells required for this diagnosis. Only one previous study specified the percentage of giant cells in the cases analyzed [6]. In agreement with our results, it reported that the mutation frequencies of *RB1* and *NF1* were significantly higher in 17 GBMs with > 30% giant cells than in TCGA *IDH*-wt GBMs [6]. Moreover, the extrapolated mutation frequencies of *RB1* and *NF1* were significantly higher in the 17 GBMs with > 30% giant cells (8/17, 47.1%; 5/17, 29.4%) than in the 18 GBMs with < 30% giant cells (2/18, 11%; 2/18, 11%) [6].

Of note, the rate of MMR genes mutations in the present GBMs (9/39 cases; 23%) was significantly higher than in the *IDH*-wt GBMs of TCGA PanCancer Atlas (1.6%) or in other cohorts of conventional GBMs of adults (3%) and children (6.6%) [33, 34]. However, in only one case MMR genes mutations were coupled with MSI, similarly to that found in TCGA PanCancer Atlas, where all 7 *MMR*-mutated GBMs lacked MSI. In our series, another GBM had MSI but lacked MMR mutations. Both GBMs with MSI had the loss of the matched MMR MSH2/MSH6 protein partners. Nevertheless, MMR losses were found in 6 additional cases with stable microsatellites. The absence of MSI in cases with the immunohistochemical loss of MMR proteins was previously reported in other cohorts of gliomas or in meningiomas [35, 36] and suggests caution in the use of immunohistochemistry for MMR proteins as a surrogate of MSI.

MMR deficiency and hypermutation are currently considered as biomarkers predictive of the response to immune checkpoint inhibition [37]. Indeed, it is reported that tumors with MMR deficiency have 10 to 100 times more somatic mutations than MMR-proficient tumors and this hypermutation state could lead to a high neoantigen load and consequent activation of the immune system and tumor destruction [38].

In the present GBMs enriched in GCs, TMB ranged between 5.4 and 153.8 (median: 9.3; inter-quartile range:8.2–12) and mutation counts were significantly higher than in the *IDH*-wt and *IDH*-mutant GBMs in TCGA PanCancer Atlas. Due to a wide TMB variability across tumor types, there is not a universal definition for hypermutation [39, 40]. Using a cut-off of ≥ 10 mutations/Mb, 16/39 (41%) of the present GBMs were hypermutated, compared to only 10/368 (2.7%) *IDH*-wt and 2/26 (7.6%) *IDH*-mutant GBMs in TCGA PanCancer Atlas. This suggests that hypermutation might represent an additional characterizing feature of GBMs enriched in GCs. In accordance, other authors reported that 1/10 (10%) GC-GBMs, analyzed by means of whole exome sequencing [10], and 2/11 (18%) GBMs with > 30% giant cells, assessed with the TML Oncomine panel, had ≥ 10 muts/Mb [6]. Moreover, most GBMs with > 100 muts/Mb had giant cell histology in other studies [31, 41].

In treatment *naïve* diffuse gliomas, hypermutation was mainly associated with *POLE* and *MMR* mutations [31, 33, 36, 42].

In agreement, one of the present hypermutated GBMs had *POLE* mutation and 8 had mutations in *MMR* genes. Of these latter, only one had MSI, suggesting that mechanisms different from defective MMR system may lead to a hypermutational status in gliomas and that the recent proposal to use MMR immunohistochemistry to identify hypermutated cases for immunotherapy should be considered with caution [43].

This study is the first to address the question on whether genetic alterations may have prognostic relevance in GC enriched GBMs. Similar to *IDH*-mutated or conventional *IDH*-wt GBMs [44], the presence of *EGFR* amplification was associated with significantly shorter patients’ survival. In contrast to that reported in gliomas treated with temozolomide [36], hypermutation was an independent predictor of longer overall survival. Of note, the OS length overlapped that of patients with *IDH*-wt GBM in TCGA PanCancer Atlas series, which might suggest that GC variant does not harbor a better prognosis than conventional *IDH-*wt GBMs. However, the subgroup of patients younger than 55 years had an OS length similar to patients with *IDH*-mutant GBM and significantly longer than patients with *IDH*-wt GBMs. This indicates that, among *IDH*-wt GBMs, giant cell variant carries a favorable prognostic significance only in younger patients.

In conclusion, the molecular landscape of GBMs with at least 30% GCs is dominated by tumor suppressor impairment represented by alterations in *TP53/MDM2* and *RB1/CDKN2A/CDK4* pathways, associated with *EGFR* amplification in more aggressive cases. Compared to conventional *IDH-*wt GBM, this variant has higher frequency of *RB1*, *NF1* and *POLE* mutations and hypermutation. In view of these latter features, a significant proportion of GC-GBMs may be potential candidates for clinical trials with immune checkpoint inhibitors.

## Supplementary Information

**Additional file 1**: Chromosomal asset of 39 GBMs enriched in giant cells. Cases are sorted by the percentage of giant cells (cases with 30-49% giant cells are in the left part of the panel, and those with > 50% giant cells are on the right) and then by ID number. The panel summarizes copy number variation (CNV) in whole chromosomes. Consensus of chromosome CNV is represented in red for copy gain events and in blue for loss events

**Additional file 2**: List of genes included in the CORE panel and types of alterations reported.

**Additional file 3**: **Table 1**. Clinical-pathological features and Tumor mutational burden of 39 GBMs enriched in giant cells. Cases are sorted by ID number. **Table 2**: List of somatic and germline mutations identified in all samples. **Table 3**. MMR mutations, MMR immunohistochemistry, MSI and TMB in 39 giant cell enriched GBMs. Cases are arranged by TMB.

## Data Availability

Data available on request due to privacy/ethical restrictions.
