# Supplementary figures and images for: IDH-wild type glioblastomas featuring at least 30% giant cells are characterized by frequent RB1 and NF1 alterations and hypermutation
Source: Acta Neuropathol Commun. 2021 Dec 24;9:200. doi: 10.1186/s40478-021-01304-5 (PMC8709962; doi:10.1186/s40478-021-01304-5)

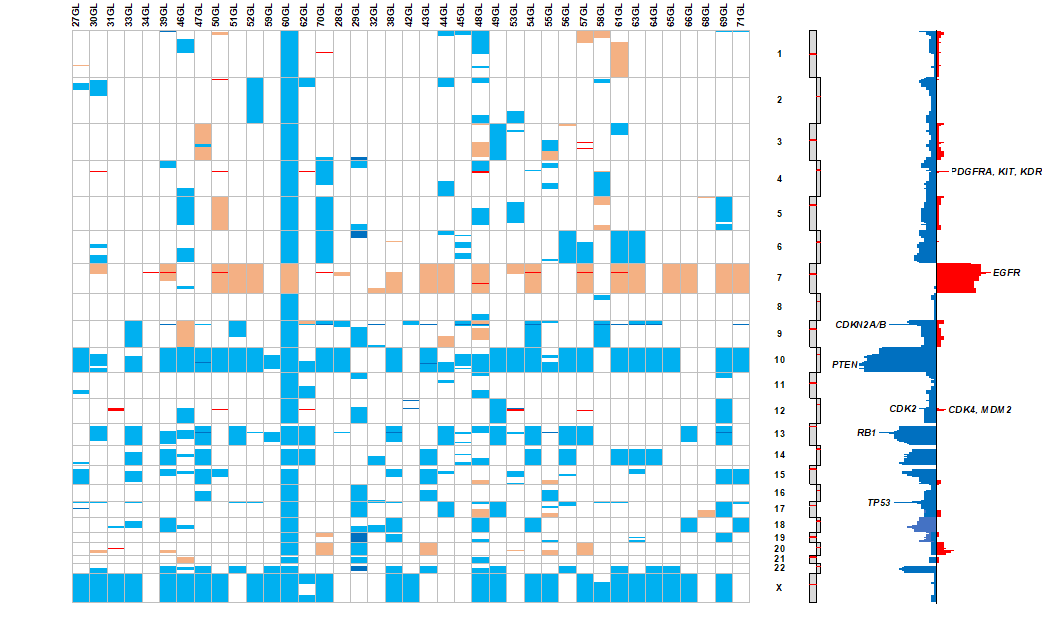

Supplement: Supplementary file 1 — Additional file 1: Chromosomal asset of 39 GBMs enriched in giant cells. Cases are sorted by the percentage of giant cells (cases with 30-49% giant cells are in the left part of the panel, and those with > 50% giant cells are on the right) and then by ID number. The panel summarizes copy number variation (CNV) in whole chromosomes. Consensus of chromosome CNV is represented in red for copy gain events and in blue for loss events [file 40478_2021_1304_MOESM1_ESM.tif]
