# Supplementary material for: IDH-wild type glioblastomas featuring at least 30% giant cells are characterized by frequent RB1 and NF1 alterations and hypermutation
Source: Acta Neuropathol Commun. 2021 Dec 24;9:200. doi: 10.1186/s40478-021-01304-5 (PMC8709962; doi:10.1186/s40478-021-01304-5)
Supplement: Supplementary file 2 — Additional file 2: List of genes included in the CORE panel and types of alterations reported. [file 40478_2021_1304_MOESM2_ESM.docx]

**Supplementary File 1. List of genes included and types of alterations reported**

|  | Genes included in CORE targeted sequencing assay and types of alterations reported | | | | | | | | |  |
| --- | --- | --- | --- | --- | --- | --- | --- | --- | --- | --- |
|  | AKT1 | B2M | CDKN1A | ERG | HIST1H3B | KMT2A | MYB | PDGFRB | RHOA | STAT5B |
|  | AKT2 | BAP1 | CDKN1B | ESR1 | HIST1H3C | KRAS | MYC | PHF6 | RNF43 | STK11 |
|  | AKT3 | *BCL2* | CDKN2A | **ETV6*** | HIST2H3C | MAP2K1 | MYCN | PIK3CA | **ROS1** | SYK |
|  | **ALK** | BLM | CDKN2B | EZH2 | HLA-A | MAP2K2 | NBN | PIK3CB | RPL5 | ***TERT*** |
|  | AMER1 | **BRAF** | CHEK2 | FAS | HLA-B | MAP2K4 | NF1 | PIK3R1 | RUNX1 | TGFBR2 |
|  | APC | BRCA1 | CIITA | FBXW7 | HLA-C | MAP3K1 | NF2 | PMS2 | SETBP1 | TP53 |
|  | APLNR | BRCA2 | CREBBP | *FGF19* | HNF1A | MAPK1 | NFE2L2 | POLE | SETD2 | TSC1 |
|  | AR | CBL | CTCF | FGFR1 | HRAS | MAX | NOTCH1 | POLQ | SF3B1 | TSC2 |
|  | ARAF | CCND1 | CTNNB1 | **FGFR2** | IDH1 | *MCL1* | NOTCH2 | PPP2R1A | SMAD4 | U2AF1 |
|  | ARID1A | *CCND2* | DAXX | **FGFR3** | IDH2 | *MDM2* | NOTCH3 | PTCH1 | SMARCA4 | VHL |
|  | ARID1B | CCND3 | DICER1 | FGFR4 | *IGF1R* | MED12 | NOTCH4 | PTEN | SMARCB1 | WT1 |
|  | ARID2 | *CCNE1* | DNMT3A | GATA3 | JAK1 | MEN1 | NPM1 | PTPN11 | SMO | *YAP1* |
|  | ASXL1 | CD274 | **EGFR** | GNA11 | JAK2 | MET | NRAS | RAC1 | SOCS1 |  |
|  | ATM | CD58 | EP300 | GNAQ | JAK3 | MLH1 | **NTRK1** | RAD21 | SPOP |  |
|  | ATR | CDK12 | EPHA3 | GNAS | JUN | MSH2 | PALB2 | RAD50 | *SRC* |  |
|  | ATRX | *CDK2* | ERBB2 | H3F3A | KDR | MSH6 | PBRM1 | **RAF1** | STAG1 |  |
|  | *AURKA* | CDK4 | ERBB3 | H3F3B | KIT | MTOR | PDCD1LG2 | RB1 | STAG2 |  |
|  | *AXL* | *CDK6* | ERBB4 | *HGF* | KLF4 | MUTYH | PDGFRA | **RET** | STAT3 |  |
|  | Coding exons \| Coding and copy number \| *Copy number only* \| Full gene footprint for structural variants \| **Fusions detected** | | | | | | | | | |
|  | * ETV6 reports NTRK3 fusions | | |  |  |  |  |  |  |  |
